# Supplementary material for: Development of a novel humanized gut-brain axis model as a tool toward personalized nutrition
Source: Commun Biol. 2026 Jan 21;9:73. doi: 10.1038/s42003-025-09472-z (PMC12824285; doi:10.1038/s42003-025-09472-z)
Supplement: Supplementary file 2 — Supplementary Information [file 42003_2025_9472_MOESM2_ESM.pdf]

Supplementary Information

Development of a novel humanized gut-brain axis model as a tool toward personalized nutrition

Myrto S Chatzopoulou[a], Ravi Vumma[b,c], Samira Prado[a,d], Mathias W Scharf[a], Victor Castro-Alves[d], Ashley N Hutchinson[a], Ignacio Rangel[a], Tatiana M Marques[a], Rebecca Wall[a], Robert J Brummer[a], Julia Rode[a,e]

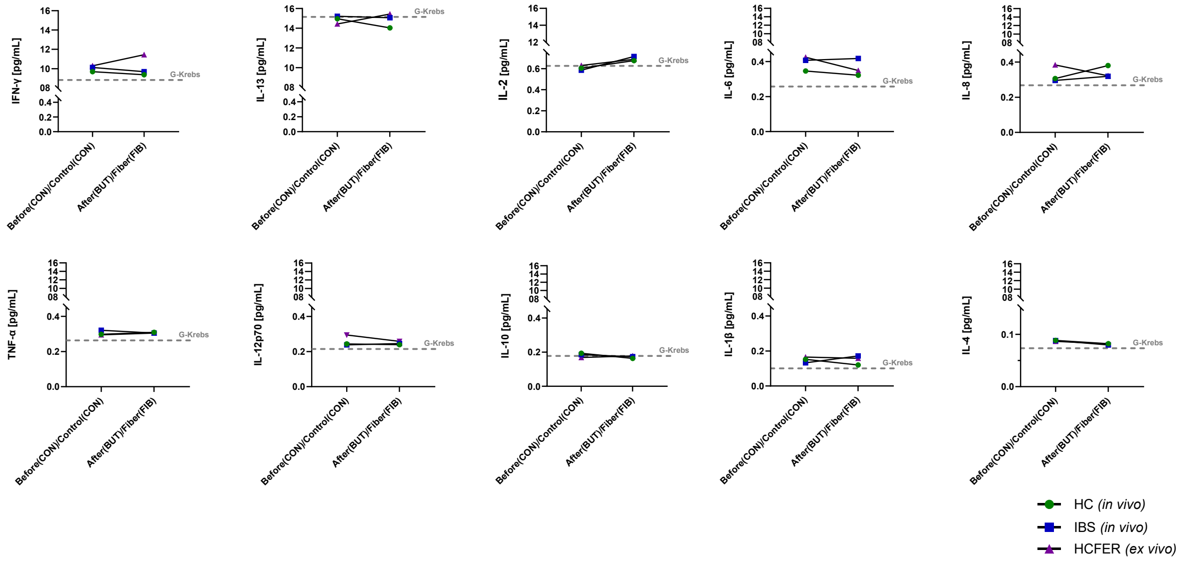

Supplementary Figure 1. Concentration of proinflammatory cytokines [pg/mL] in pooled serosal fluid samples. *(A) Interferon gamma (IFN-γ), (B) Interleukin (IL)-13, (C) IL-2, (D) IL-6, (E) IL-8, (F) Tumor-necrosis factor alpha (TNF-α), (G) IL-12p70, (H) IL-10, (I) IL-1β, and (J) IL-4. Presented from left to right are the control exposures as "Before", meaning before in vivo butyrate infusion (HC-CON/IBS-CON), or "Control", meaning ex vivo exposure with the control fecal fermentation supernatant (HC-FERCON); and the treatment exposures as "After" for after butyrate infusion (HC-BUT/IBS-BUT), or "Fiber" for the exposure to the fecal fermentation with fiber (HC-FERFIB). The grey dashed line represents the concentration measured in the physiological serosal carrier-solution (G-Krebs). Green circles present HC-CON and HC-BUT, blue squares present IBS-CON and IBS-BUT, purple triangle presents HC-FERCON and HC-FERFIB. Cytokines of pooled serosal fluids were assessed without replicates, hence every datapoint presents a single measure.*

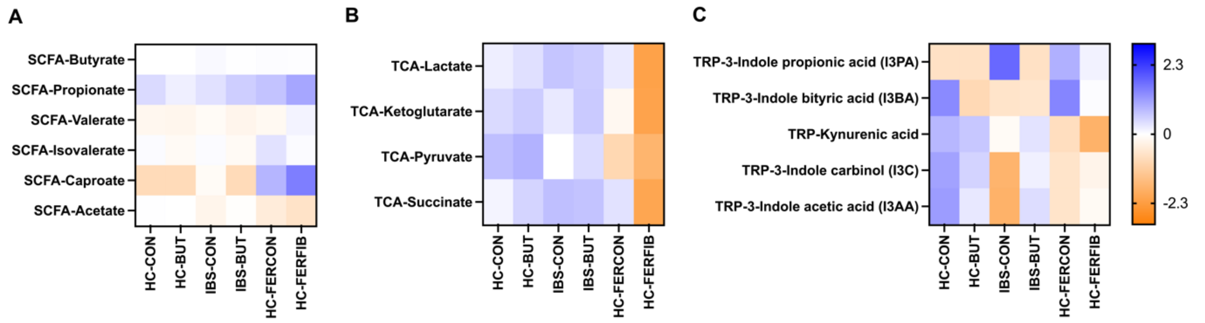

Supplementary Figure 2. Metabolite profiles of serosal fluids. *(A) short-chain fatty acids (SCFAs), (B) tricarboxylic acid (TCA) cycle-related metabolites, and (C) tryptophan (TRP) metabolism-related metabolites, of serosal fluids presented as log2 fold changes against their carrier solution G-Krebs. A positive value (blue) indicates higher levels in the respective serosal fluid than G-Krebs, and vice versa (orange). X-axis presents exposure to serosal fluid pool derived from healthy biopsies collected from the unexposed colon (HC-CON), from healthy biopsies collected after in vivo butyrate exposure (HC-BUT), from irritable bowel syndrome biopsies collected from the unexposed colon (IBS-CON), from IBS biopsies collected after in vivo butyrate exposure (IBS-BUT), from healthy biopsies ex vivo exposed to supernatant of fecal control fermentation (HC-FERCON), and from healthy biopsies ex vivo exposed to supernatant of fecal fiber fermentation (HC-FERFIB).*

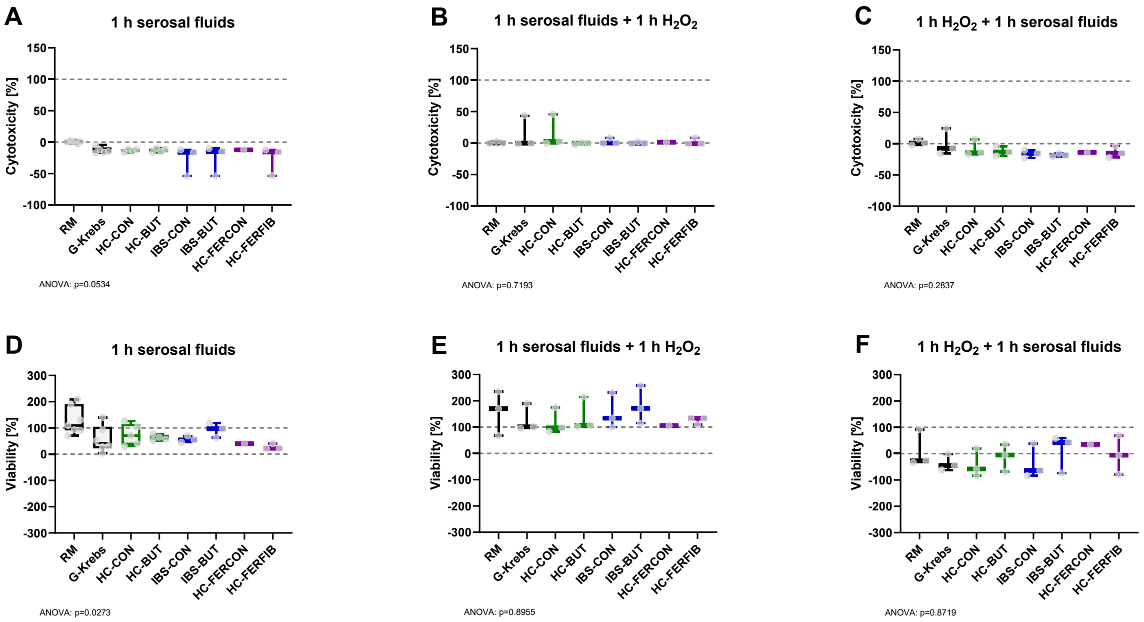

Supplementary Figure 3. None of the acute (1 h) experimental conditions was cytotoxic (A-C) or significantly hampered viability (D-F) compared to exposure to G-Krebs. *(A&D) 1 h exposure to serosal fluids alone, (B&E) exposure to 1 h serosal fluids as preventive measure before oxidatively stressing the cells (by 10 µM H_2O_2), and (C&F) exposure to 1 h serosal fluids as a treatment measure after oxidatively stressing the cells. X-axis presents exposure to regular medium (RM), serosal fluid carrier solution G-Krebs, and serosal fluid pool derived from healthy biopsies collected from the unexposed colon (HC-CON), from healthy biopsies collected after in vivo butyrate exposure (HC-BUT), from irritable bowel syndrome biopsies collected from the unexposed colon (IBS-CON), from IBS biopsies collected after in vivo butyrate exposure (IBS-BUT), from healthy biopsies ex vivo exposed to supernatant of fecal control fermentation (HC-FERCON), and from healthy biopsies ex vivo exposed to supernatant of fecal fiber fermentation (HC-FERFIB). Per condition n≤7 biologically independent experiments. Data visualized as boxplots, whiskers indicated minimum to maximum, individual data points are overlayed. Black boxplots present control conditions RM and G-Krebs, green presents HC-CON and HC-BUT, blue presents IBS-CON and IBS-BUT, purple presents HC-FERCON and HC-FERFIB. One-way ANOVA with posthoc uncorrected Fisher's LSD multiple comparisons test versus G-Krebs control condition. Exact p-value for ANOVA and if significant for posthoc tests provided in figure.*

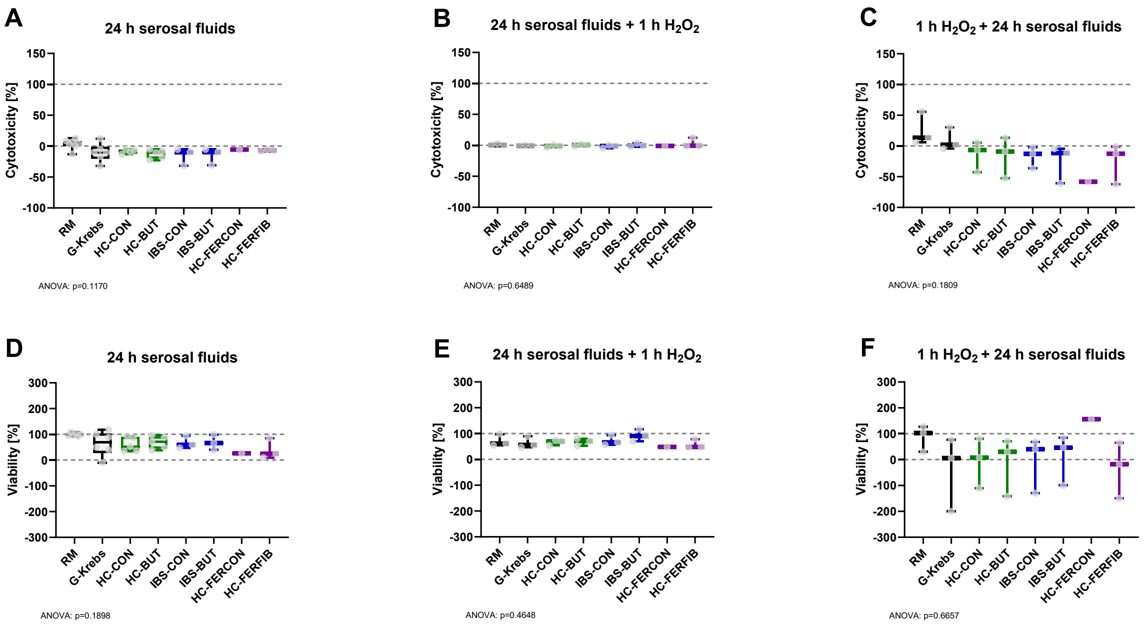

Supplementary Figure 4. None of the relatively extended (24 h) experimental conditions was cytotoxic (A-C) or significantly hampered viability (D-F) compared to exposure to G-Krebs. *(A&D) 24 h exposure to serosal fluids alone, (B&E) exposure to 24 h serosal fluids as preventive measure before oxidatively stressing the cells (by 10 µM H_2O_2), and (C&F) exposure to 24 h serosal fluids as a treatment measure after oxidatively stressing the cells. X-axis presents exposure to regular medium (RM), serosal fluid carrier solution G-Krebs, and serosal fluid pool derived from healthy biopsies collected from the unexposed colon (HC-CON), from healthy biopsies collected after in vivo butyrate exposure (HC-BUT), from irritable bowel syndrome biopsies collected from the unexposed colon (IBS-CON), from IBS biopsies collected after in vivo butyrate exposure (IBS-BUT), from healthy biopsies ex vivo exposed to supernatant of fecal control fermentation (HC-FERCON), and from healthy biopsies ex vivo exposed to supernatant of fecal fiber fermentation (HC-FERFIB). Per condition n≤7 biologically independent experiments. Data visualized as boxplots, whiskers indicated minimum to maximum, individual data points are overlayed. Black boxplots present control conditions RM and G-Krebs, green presents HC-CON and HC-BUT, blue presents IBS-CON and IBS-BUT, purple presents HC-FERCON and HC-FERFIB. One-way ANOVA with posthoc uncorrected Fisher's LSD multiple comparisons test versus G-Krebs control condition. Exact p-value for ANOVA and if significant for posthoc tests provided in figure.*

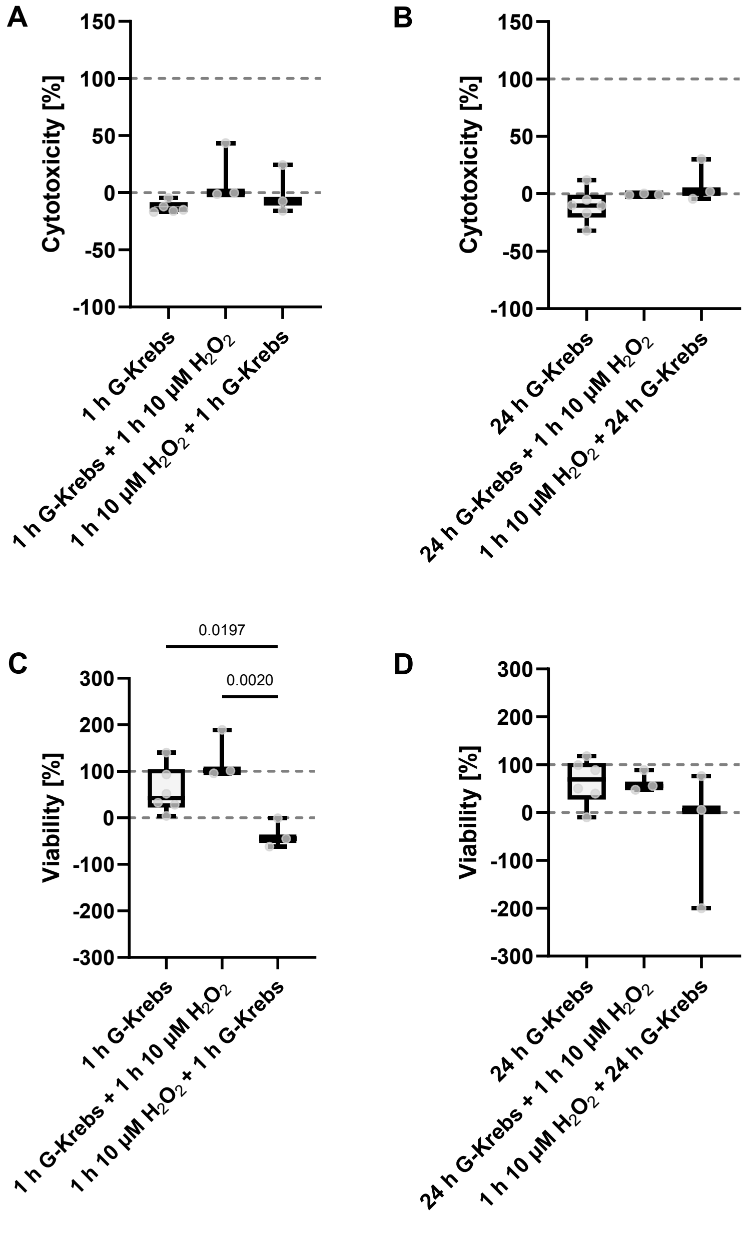

Supplementary Figure 5. Validation of H_2O_2 as oxidative stressor in the setting of this project. *While incubation with 10 µM H_2O_2 for 1 h was non-cytotoxic (A&B), metabolic activity was somewhat hampered (C&D). No statistical comparisons were made. Data visualized as boxplots, whiskers indicated minimum to maximum, individual data points are overlayed.*

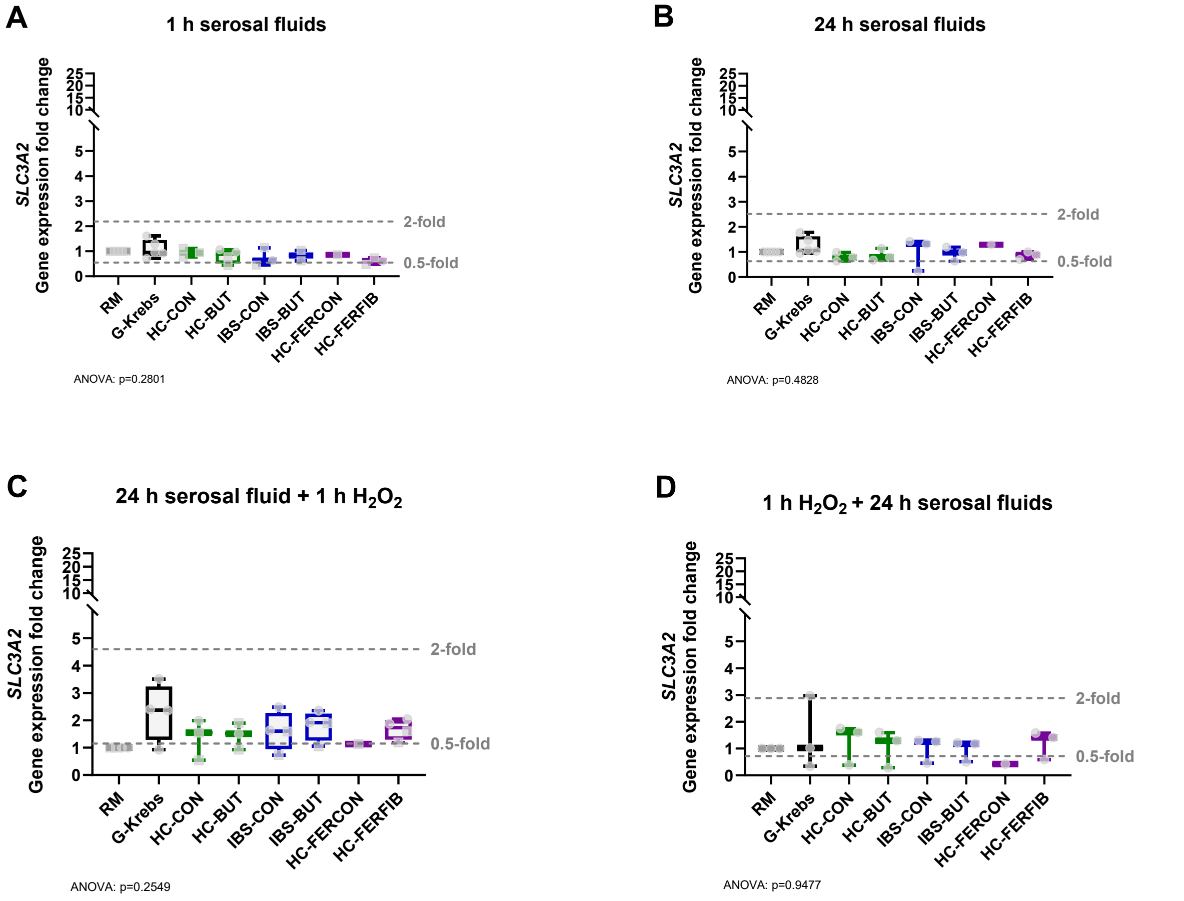

Supplementary Figure 6. Gene expression of the common subunit SLC3A2 of the major and minor tryptophan transporters LAT1 and LAT2 as well as other amino acid transporters, normalized to reference gene GAPDH and the methodological reference condition regular medium. *(A) 1 h exposure to serosal fluids alone, (B) 24 h exposure to serosal fluids alone, (C) exposure to 24 h serosal fluids as preventive measure before oxidatively stressing the cells (by 10 µM H_2O_2), and (D) exposure to 24 h serosal fluids as a treatment measure after oxidatively stressing the cells. X-axis presents exposure to regular medium (RM), serosal fluid carrier solution G-Krebs, and serosal fluid pool derived from healthy biopsies collected from the unexposed colon (HC-CON), from healthy biopsies collected after in vivo butyrate exposure (HC-BUT), from irritable bowel syndrome biopsies collected from the unexposed colon (IBS-CON), from IBS biopsies collected after in vivo butyrate exposure (IBS-BUT), from healthy biopsies ex vivo exposed to supernatant of fecal control fermentation (HC-FERCON), and from healthy biopsies ex vivo exposed to supernatant of fecal fiber fermentation (HC-FERFIB). Per condition n≤5 biologically independent experiments. Data visualized as boxplots, whiskers indicated minimum to maximum, individual data points are overlayed. Black boxplots present control conditions RM and G-Krebs, green presents HC-CON and HC-BUT, blue presents IBS-CON and IBS-BUT, purple presents HC-FERCON and HC-FERFIB. Commonly used thresholds for biological relevance indicated by the grey dashed lines for 0.5- and 2-fold changes compared to G-Krebs. One-way ANOVA with posthoc uncorrected Fisher's LSD multiple comparisons test versus G-Krebs control condition. Exact p-value for ANOVA and if significant for posthoc tests provided in figure.*

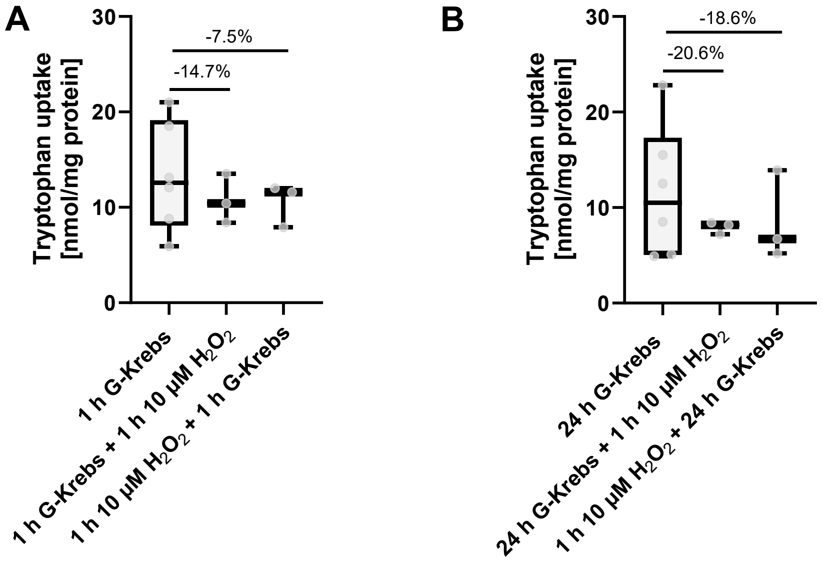

***Supplementary Figure 7. Validation of H_2O_2 as oxidative stressor in the setting of this project based on the first experimental batch**. Incubation with 10 µM H_2O_2 decreased tryptophan uptake by more than 10% on average. Data visualized as boxplots, whiskers indicated minimum to maximum, individual data points are overlayed, individual data points are overlayed.*

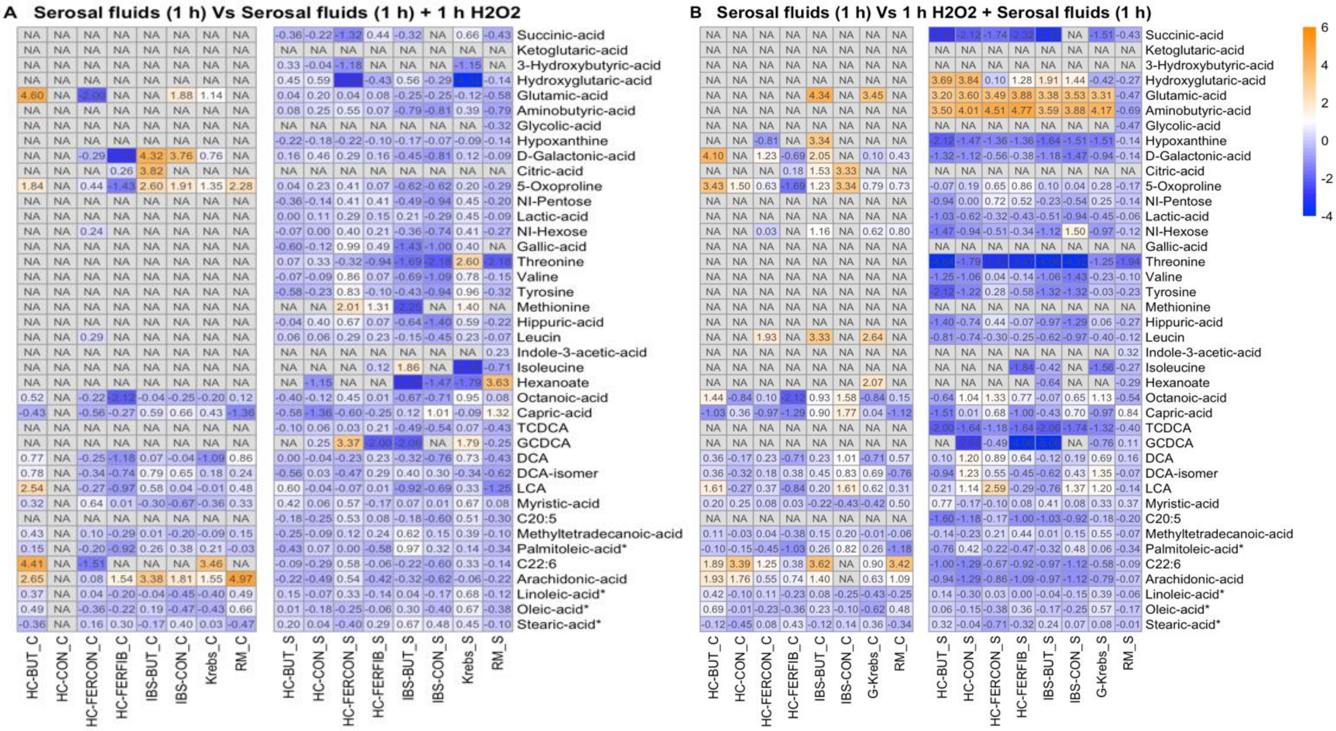

***Supplementary Figure 8. Metabolite profiles of cell harvests, shown on the left of each panel (annotated as "C"); and supernatants, shown on the right of each panel (annotated as "S").** Results are presented as log2 fold changes of 1 h exposure with serosal fluids alone against (A) the preventive measure (1 h serosal fluids and then 1 h H_2O_2) or (B) the treatment measure (1 h H_2O_2 and then 1 h serosal fluids). X-axis presents exposure to regular medium (RM), serosal fluid carrier solution G-Krebs, and serosal fluid pool derived from healthy biopsies collected from the unexposed colon (HC-CON), from healthy biopsies collected after in vivo butyrate exposure (HC-BUT), from irritable bowel syndrome biopsies collected from the unexposed colon (IBS-CON), from IBS biopsies collected after in vivo butyrate exposure (IBS-BUT), from healthy biopsies ex vivo exposed to supernatant of fecal*

control fermentation (HC-FERCON), and from healthy biopsies ex vivo exposed to supernatant of fecal fiber fermentation (HC-FERFIB). NA – metabolite concentration below detection limit in any of the two samples; all metabolites were below detection limit for the "preventive" sample from HC-CON cells (HC-CON_C). A positive value (orange) indicates higher levels in the respective test condition (preventive or treatment) than serosal fluid exposure alone, and vice versa (blue).

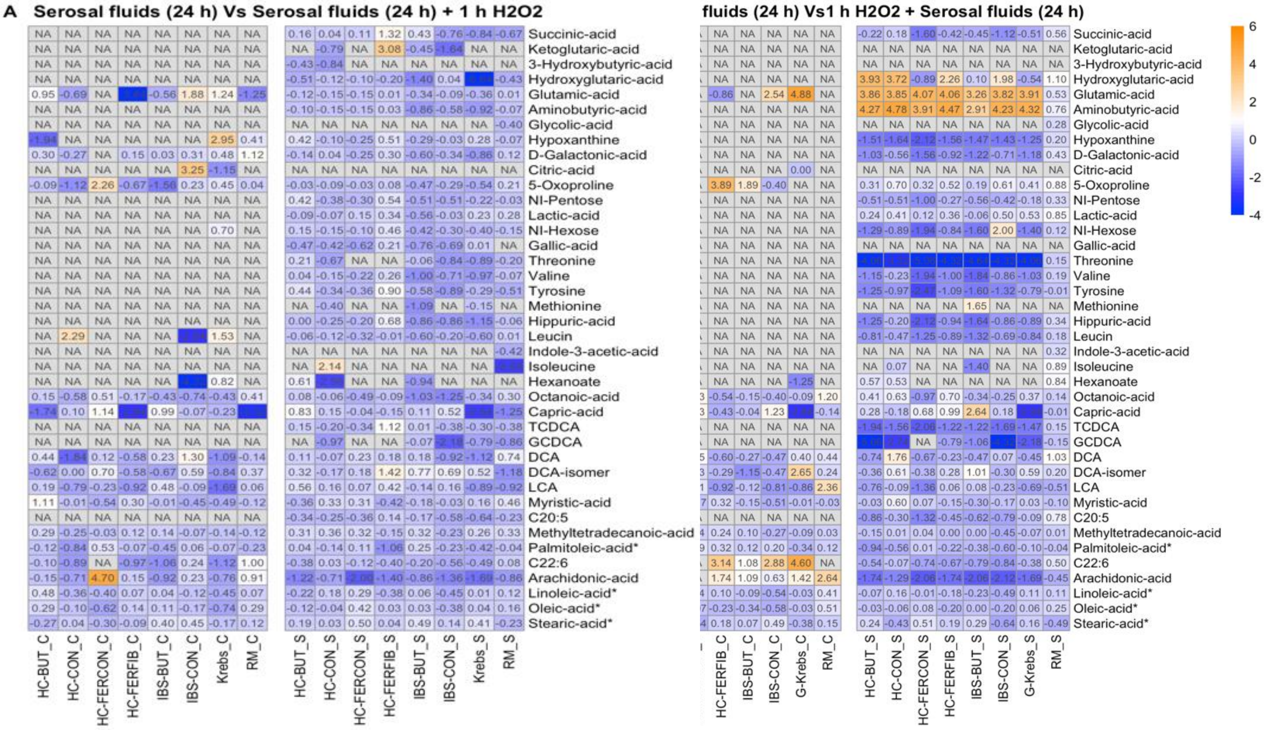

Supplementary Figure 9. Metabolite profiles of cell harvests, shown on the left of each panel (annotated as "C"); and supernatants, shown on the right of each panel (annotated as "S"). *Results are presented as log2 fold changes of 24 h exposure with serosal fluids alone against (A) the preventive measure (1 h serosal fluids and then 1 h H_2O_2) or (B) the treatment measure (1 h H_2O_2 and then 1 h serosal fluids). X-axis presents exposure to regular medium (RM), serosal fluid carrier solution G-Krebs, and serosal fluid pool derived from healthy biopsies collected from the unexposed colon (HC-CON), from healthy biopsies collected after in vivo butyrate exposure (HC-BUT), from irritable bowel syndrome biopsies collected from the unexposed colon (IBS-CON), from IBS biopsies collected after in vivo butyrate exposure (IBS-BUT), from healthy biopsies ex vivo exposed to supernatant of fecal control fermentation (HC-FERCON), and from healthy biopsies ex vivo exposed to supernatant of fecal fiber fermentation (HC-FERFIB). NA – metabolite below detection limit in any of the two samples. A positive value (orange) indicates higher levels in the respective test condition (preventive or treatment) than serosal fluid exposure alone, and vice versa (blue).*

Supplementary Table 1. Dynamic range of proinflammatory cytokines. *Concentration is measured in the calibrators as pg/mL and values are presented as a mean of triplicates.*

**Proinflammatory cytokine**	**Dynamic range [pg/mL]**
IFN-γ	0.39-1374.12
IL-13	0.64-541.77
IL-2	0. 36-1512.11
IL-6	0.13-745.30
IL-8	0.14-586.45
TNF-α	0.09-375.94
IL-12p70	0.12-447.72
IL-10	0.09-380.59
IL-1β	0.16-630.19
IL-4	0.06-234.4

Abbreviations: IFN = interferon, IL = interleukin, TNF = tumor necrosis factor

Supplementary Table 2. UHPLC-qToF-HRMS analysis performance of serosal fluids after derivatization with 3-nitrophenyl hydrazine (3-NPH). *Accurate mass-to-charge ratio (m/z) of the 3-NPH derivatives, retention time (RT) and average relative standard deviation (%RSD) of internal standards in the quality control (QC, n=4) and samples (n=7).*

**Internal standards**	**m/z**	**RT (min)**	**%RSD**
Acetate-d4	197.0756	3.33	8.7
Propionate-d8	210.0850	3.88	7.9
Butyrate-d8	229.1321	4.28	7.6
Succinate-d4	391.1306	4.40	7.6

Supplementary Table 3. UHPLC-qToF-HRMS analysis performance of cell harvest and supernatants. *Accurate mass-to-charge ratio (m/z), retention time (RT) and average relative standard deviation (%RSD) of internal standards in the quality control (QC, n=4) and samples (n=96).*

Internal standards	m/z	RT (min)	%RSD
Succinate-d4	121.0439	0.53	26.9
Tryptophan-d5	208.1136	0.87	12.6
Hexanoic acid d3	118.0947	1.28	8.0
GUDCA-d4	452.3316	3.54	8.2
UDCA-d4	395.3100	3.96	7.1
GCA-d4	468.3265	3.98	7.9
CA-d4	411.3051	4.36	7.2
GDCA-d4	452.3317	4.53	8.1
CDCA-d4	395.3100	4.99	8.1
DCA-d4	395.3101	5.10	8.0
GLCA-d4	436.3366	5.12	8.9
TCA-d4	518.3396	5.12	15.2
LCA-d4	379.3151	5.61	7.8
Heptadecanoic acid	269.2485	6.99	22.7

Abbreviations: GUDCA = glycoursodeoxycholic acid, UDCA = ursodeoxycholic acid, GCA = glycocholic acid, CA = cholic acid, GDCA = glycodeoxycholic acid, CDCA = chenodeoxycholic acid, DCA = deoxycholic acid, GLCA = glucuronic acid, TCA = tricarboxylic acid, LCA = lithocholic acid.

Supplementary Table 4. Complete list of all analyzed and hence putatively detectable metabolites in any sample type. *Results of such are only presented if detected in any sample.*

Chemical Name	Subclass	Molecular Formula	Monoisotopic mass (Da)
5-Oxoproline	Amino acids and derivates	C5H7NO3	129.0426
Aminobutyric Acid	Amino acids and derivates	C4H9NO2	103.0633
Glutamic Acid	Amino acids and derivates	C5H9NO4	147.0532
Isoleucine	Amino acids and derivates	C6H13NO2	131.0946
Leucine	Amino acids and derivates	C6H13NO2	131.0946
Methionine	Amino acids and derivates	C5H11NO2S	149.0510

Threonine	Amino acids and derivates	C4H9NO3	119.0582
Tyrosine	Amino acids and derivates	C9H11NO3	181.0739
Valine	Amino acids and derivates	C5H11NO2	117.0790
Gallic Acid	Benzoic acids and derivates	C7H6O5	170.0215
Hippuric Acid	Benzoic acids and derivates	C9H9NO3	179.0582
DCA	Bile acids and derivates	C24H40O4	392.2927
DCA isomer	Bile acids and derivates	C24H40O4	392.2927
GCDCA	Bile acids and derivates	C26H43NO5	449.3141
LCA	Bile acids and derivates	C24H40O3	376.2977
TCDCA	Bile acids and derivates	C26H45NO6S	499.2968
Indole-3-Acetic Acid	Indoles and derivates	C10H9NO2	175.0633
Indole-3-Butyric Acid	Indoles and derivates	C12H13NO2	203.0946
Indole-3-Carbinol	Indoles and derivates	C9H9NO	147.0684
Indole-3-Propionic Acid	Indoles and derivates	C11H11NO2	189.0790
Kinurenic Acid	Indoles and derivates	C10H7NO3	189.0426
Arachidonic Acid	Long-chain fatty acids	C20H32O2	304.2402
Decanoic Acid (Capric Acid)	Long-chain fatty acids	C10H20O2	172.1463
Docosahexaenoic Acid	Long-chain fatty acids	C22H32O2	328.2402
Eicosapentaenoic Acid	Long-chain fatty acids	C20H30O2	302.2246
Hexanoate	Long-chain fatty acids	C6H12O2	116.0837
Hexanoic acid	Long-chain fatty acids	C6H12O2	116.0837
Linoleic Acid	Long-chain fatty acids	C18H32O2	280.2402
Methyltetradecanoic Acid	Long-chain fatty acids	C15H30O2	242.2246
Myristic Acid	Long-chain fatty acids	C14H28O2	228.2089
Octanoic Acid	Long-chain fatty acids	C8H16O2	144.1150
Oleic Acid	Long-chain fatty acids	C18H34O2	282.2559

Palmitoleic Acid	Long-chain fatty acids	C16H30O2	254.2246
Stearic Acid	Long-chain fatty acids	C18H36O2	284.2715
Galactonic Acid	Monosaccharides and derivates	C6H12O7	196.0583
NI Hexose (glucose)	Monosaccharides and derivates	C6H12O6	180.0634
NI Pentose (ribose)	Monosaccharides and derivates	C5H10O5	150.0528
Citric Acid	Organic acids and derivates	C6H8O7	192.0270
Glycolic Acid	Organic acids and derivates	C2H4O3	76.0160
Hydroxyglutaric Acid	Organic acids and derivates	C5H8O5	148.0372
Ketoglutaric Acid	Organic acids and derivates	C5H6O5	146.0215
Lactic Acid	Organic acids and derivates	C3H6O3	90.0317
Pyruvic acid	Organic acids and derivates	C3H4O3	88.0160
Succinic Acid	Organic acids and derivates	C4H6O4	118.0266
Hypoxanthine	Purines and purine derivates	C5H4N4O	136.0385
3-Hydroxybutyric Acid	SCFA and related compounds	C4H8O3	104.0473
Acetic acid	SCFA and related compounds	C2H4O2	60.0211
Butyric acid	SCFA and related compounds	C4H8O2	88.0524
Isopentanoic acid	SCFA and related compounds	C5H10O2	102.0681
Pentanoic acid	SCFA and related compounds	C5H10O2	102.0681
Propionic acid	SCFA and related compounds	C3H6O2	74.0368

Abbreviations: DCA = deoxycholic acid, GCDCA = glycochenodeoxycholic acid, LCA = lithocholic acid, TCDCA = taurochenodeoxycholic acid.

Supplementary Note 1. Discussion of technical aspects

Evidence supports that cell responses highly depend on the length and concentration of the H_2O_2 exposure but also on the age and type of the cells used [1]. In a recent study, Pieńkowska and collaborators showed that the half-life of 50 µM H_2O_2 in the presence of 5 x 10^3 H8F2p25LM skin fibroblast cells is approximately 61.5 minutes [2]. Furthermore, Ma et al. found that H_2O_2 in different concentrations could differentially influence the production of reactive oxygen species (ROS) and even lead to contradicting effects on cell viability with lower concentrations (5-15 µM) promoting cell proliferation instead of hindering it [1]. In our study (see Supplementary Figure 5), the concentration of 10 µM was found non-cytotoxic and it successfully compromised viability (metabolic activity) when it was used as the initial exposure (before incubation with serosal fluids). In addition, it caused detrimental effects in tryptophan uptake (see Supplementary Figure 7), which were comparable to our previously published work, where the average decrease from four presented sets of experiments was 10.8% [3].

Pro-inflammatory processes and generation of ROS are known to compromise blood-brain barrier (BBB) integrity, which could negatively impact the expression of large amino acid transporters LAT1 and LAT2 and eventually, the uptake of tryptophan (Supplementary Figure 7), thereby increasing the risk for neurobehavioral disorders [4].

Several of the outcome measures were based on absorbance measures. Since the regular medium contained phenol red which was diluted when adding G-Krebs or serosal fluids, all comparisons were performed against the G-Krebs control. Regular medium was instead used as a methodological reference.

Furthermore, we strived to hold a balance of regular medium and serosal fluids to have enough active substances while not depriving the cells from nutrients. Also, the available total volume of serosal fluids was limited, hence samples were pooled across subjects.

The cytokine results are to be interpreted with caution. To note is that all ten measured cytokines were detectable in all sample types including G-Krebs solution, which was somewhat unexpected. To date, it is unclear whether ubiquitarian factors, such as for example the salts present in G-Krebs solution and

hence also all serosal fluids, may have interfered with the electrical signal during plate reading. Contrarily, however, serosal fluids collected from 90-minute Ussing chambers with colonic biopsies of healthy adults exposed to fecal fiber fermentation supernatants with *Pleurotus erynii* whole food matrix did not contain any detectable cytokine levels, in a recent study [5]. While the experimental procedure was very similar to the herein reported one, the cytokine panel did overlap, but was not identical to the herein tested, the utilized MSD assay was less sensitive and samples were diluted 1:1 – which all may have contributed to the discrepancy in results.

Due to experiencing a generally low performance of the currently commercially available tests for LAT1 and LAT2, we are cautious in interpreting those results.

Failing to capture effects when measuring immediately after the 1 h exposure periods, could also be a possibility, which is also supported by the results of 24 h exposures seemingly more stable generally, though normally sudden changes in the cells' environment would provoke rapid adaptation within seconds or minutes [6]. Another factor might be lower confluency, hence cell numbers, in the 1 h experiments compared to the 24 h experiments where cells were cultured one extra day.

The variation in the LDH assay results is smaller than in the AlamarBlue assay results, possibly due to more dependency on the actual cell number in the latter. Protein expression might take longer to be affected than gene expression.

Also, the H_2O_2 effect may be only initiated in the first 1 h but longer lasting and then the addition of serosal fluids may also be an additional stressor (while it is protective when opposite). H_2O_2 might also silence the cells temporarily metabolically (in 1 h) but not for the following 24 h.

BUT represents a longer (90 min) *in vivo* exposure with butyrate which occurs quite some time before the serosal fluids are collected (at least 2-3 h after end of exposure). FER represents an *ex vivo* exposure with fecal fiber fermentation supernatants all the time until serosal fluids are collected (90 min).

Supplementary References

1 Ma, L. et al. H2O2 inhibits proliferation and mediates suppression of migration via DLC1/RhoA signaling in cancer cells. Asian Pac J Cancer Prev 16, 1637-1642 (2015). https://doi.org/10.7314/apjcp.2015.16.4.1637

2 Pienkowska, N. et al. Effect of antioxidants on the H2O2-induced premature senescence of human fibroblasts. Aging-Us 12, 1910-1927 (2020). https://doi.org/10.18632/aging.102730

3 Rode, J. et al. Butyrate Rescues Oxidative Stress-Induced Transport Deficits of Tryptophan: Potential Implication in Affective or Gut-Brain Axis Disorders. Neuropsychobiology 80, 253-263 (2021). https://doi.org/10.1159/000510886

4 Jenkins, T. A., Nguyen, J. C., Polglaze, K. E. & Bertrand, P. P. Influence of Tryptophan and Serotonin on Mood and Cognition with a Possible Role of the Gut-Brain Axis. Nutrients 8 (2016). https://doi.org/10.3390/nu8010056

5 Kerezoudi, EN. et al. Pleurotus eryngii Mushrooms Fermented with Human Fecal Microbiota Protect Intestinal Barrier Integrity: Immune Modulation and Signalling Pathways Counter Deoxycholic Acid-Induced Disruption in Healthy Colonic Tissue. Nutrients Feb 14;17(4):694. (2025). https://doi.org/10.3390/nu17040694.

6 Sthijns, M. M., Weseler, A. R., Bast, A. & Haenen, G. R. Time in Redox Adaptation Processes: From Evolution to Hormesis. Int J Mol Sci 17 (2016). https://doi.org/10.3390/ijms17101649
