# Supplementary material for: Development of a novel humanized gut-brain axis model as a tool toward personalized nutrition
Source: Commun Biol. 2026 Jan 21;9:73. doi: 10.1038/s42003-025-09472-z (PMC12824285; doi:10.1038/s42003-025-09472-z)
Supplement: Supplementary file 3 — Description of Additional Supplementary Materials [file 42003_2025_9472_MOESM3_ESM.pdf]

Description of Additional Supplementary Files

File name: Supplementary Data 1

Description: Numeric source data for Figures 1-6
